# Loss of Circulating CD8+ CD161^high^ T Cells in Primary Progressive Multiple Sclerosis

**DOI:** 10.3389/fimmu.2019.01922

**Published:** 2019-08-14

**Authors:** Massimo Acquaviva, Claudia Bassani, Nicole Sarno, Gloria Dalla Costa, Marzia Romeo, Francesca Sangalli, Bruno Colombo, Lucia Moiola, Vittorio Martinelli, Giancarlo Comi, Cinthia Farina

**Affiliations:** Division of Neuroscience, Institute of Experimental Neurology, San Raffaele Scientific Institute, Milan, Italy

**Keywords:** CD161, CD8, blood, progressive multiple sclerosis, MAIT cells

## Abstract

Recent evidence suggests that the primary progressive form of multiple sclerosis (PP-MS) may present with specific immunological alterations. In this study we focused our attention on CD161, an NK and T cell marker upregulated in relapsing-remitting MS, and investigated its transcript and protein levels in blood cells from PP-MS and healthy individuals. We demonstrated transcriptional downregulation of CD161 in PP-MS and described concomitant mRNA reduction for RORgt, CCR6, CXCR6, KLRK1/NKG2D and many other markers typical of mucosa associated invariant T (MAIT) cells. Targeted multiparametric flow cytometry on fresh blood cells from an independent cohort of case-control subjects confirmed the selective loss of circulating CD8 CD161^high^ T cells, which consist mainly of MAIT cells, and not of CD8 CD161^int^ T cells in PP-MS. These data demonstrate alterations in a specific circulating immune cell subset in MS patients with progressive onset.

## Introduction

Multiple sclerosis (MS) is a chronic inflammatory disorder of the central nervous system (CNS), presenting with distinct clinical courses (1). Most of the patients display the relapsing-remitting (RR) form of disease, where episodes of neurological deterioration due to CNS inflammation and demyelination are followed by partial or total recovery of functions and remission. A minority of the patients experience progressive deterioration of disability due to demyelination and neurodegeneration with unfrequent neuroradiological evidence of immune cell infiltration into the CNS, and are referred to as primary progressive (PP) MS patients (1). The study of the pathogenic processes occurring during experimental neuroinflammation has led to a large repertoire of drugs targeting peripheral immunity, most of which have shown efficacy in treating the relapsing-remitting form of disease but not progressive MS (2). These observations have led to the hypothesis that the contribution of the immune system to PP-MS is not relevant. However, the evidence that therapeutic depletion of CD20 positive B lymphocytes can be of benefit in progressive MS (2) has reproposed the need of understanding the immunological alterations associated with this course of disease. This information may lay the basis for novel knowledge-driven therapeutic immune checkpoints for each MS stage. As a first step in this direction, we have investigated blood transcriptomics changes in MS and recently demonstrated that peripheral blood mononuclear cells (PBMC) carry important transcriptional information whose monitoring may indeed emphasize dysregulations in genes and pathways at specific stages of MS (3, 4). In this paper we focused our attention on transcript and protein levels of CD161, also called KLRB1, a C-type lectin expressed by NK cells as well as T lymphocytes. CD161 may act as costimulatory molecule in the context of T cell receptor mediated activation (5) and regulate transendothelial migration of T lymphocytes (6). It is an activating receptor during NK cell lineage development (7) and a marker of human IL17-producing T cells (8) upregulated in relapsing-remitting MS (9). Here we show selective loss of circulating CD8+ CD161^high^ T cells and not of CD8+ CD161^int^ T cells in PP-MS.

## Materials and Methods

### Human Subjects

Investigations were conducted according to the principles expressed in the Declaration of Helsinki and after approval of the study by the Ethics Committee of Ospedale San Raffaele. Peripheral blood was drawn after signing of the informed consent. MS subjects were diagnosed according to McDonald criteria (10), and were not under immunomodulatory/immunosuppressive therapy for MS. Demographic and clinical data of enrolled patients and controls for transcriptomics or targeted flow cytometry studies are listed in Table 1 of (3) and Supplementary Table 1 of the current manuscript, respectively.

### Peripheral Blood Mononuclear Cell (PBMC) Preparation and Flow Cytometry

PBMC were isolated using a discontinuous density gradient (Lymphoprep, Nycomed, Oslo, Norway) as already described (3, 4, 11). Viable cells were counted by Trypan Blue (Sigma-Aldrich, Milan, Italy) exclusion. Freshly prepared cells were stained with three distinct mixes of antibodies: the first included FITC-labeled anti-human CD56 (NCAM16.2, BD Biosciences) and PE-labeled anti-human CD161 (HP-3G10, Biolegend), the second FITC-labeled anti-human CD19 (HIB19, Biolegend) and PE-labeled anti-human CD161 (HP-3G10, Biolegend), the third Pacific Blue-labeled anti-human CD3 (UCHT1, Biolegend), PerCP-labeled anti-human CD4 (SK3 Biolegend), APC-H7-labeled anti-human CD8 (SK1, BD Biosciences), FITC-labeled anti-human CD45RO (UCHL1, Biolegend), PeCy7-labeled anti-human CD197 (CCR7, BD) and PE-labeled anti-human CD161 (HP-3G10, Biolegend). Samples were acquired at FACSCanto II using FACS DIVA software (all from BD Biosciences). Data were analyzed by FlowJo software (FlowJo LLC). Gating strategy and representative results are shown in Supplementary Figure 1. Thresholds were set on FMO and isotype controls.

### Transcriptomics Analysis

The human PBMC transcriptomics dataset analyzed in this study was recently published (3, 4) and deposited at EBI Array express database (ID: E-MTAB-4890). This dataset was generated by Illumina Human Ref-8 v2 microarrays and included PBMC transcriptomes of 23 PP-MS and 40 healthy controls (HC). Raw data were processed in R using Limma package. Background was subtracted by nec method, data were normalized using cubic spline procedure, and batch effects were corrected by Combat. Probes correlating with age in the healthy population were removed. Probes with a mean intensity value lower than 100 in all experimental groups were filtered out.

### Statistical Analysis

Normality of data distribution was assessed by D'Agostino and Pearson statistics. Unpaired *t*-test (in case of normal distribution) or non-parametric Mann–Whitney *U*-test (in case of non-normal distribution) was performed to compare means between independent groups. Welch's correction was applied to the *t*-test in case of significantly different variances. All the *p*-values were two-sided and subjected to a significance threshold of 0.05.

## Results

**Figure 1:**
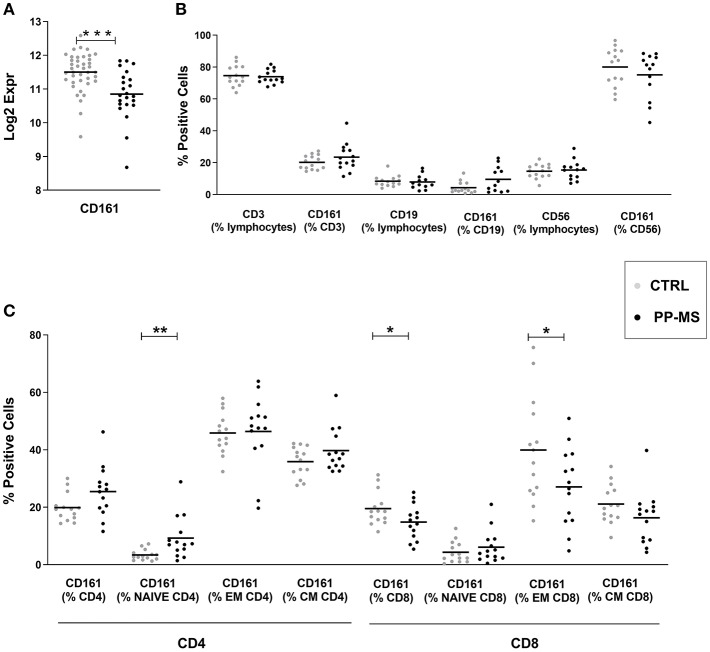
CD161 transcript and protein levels in PBMC of primary progressive MS and healthy subjects. **(A)** CD161 transcript levels in PBMC from a first cohort of PP-MS and healthy individuals. **(B)** Frequency of CD161 positive cells in distinct immune cell subsets in a second independent case-control cohort. **(C)** Frequency of CD161 positive cells in naïve, central memory (CM) and effector memory (EM) CD4 or CD8 T lymphocytes from the second cohort of subjects. ^*^p < 0.05, ^**^p < 0.01, ^***^p < 0.001.

Transcriptional profiles of peripheral blood mononuclear cells (PBMC) from 40 healthy subjects and 23 primary progressive multiple sclerosis patients were retrieved from our transcriptomics dataset published in Srinivasan et al. (3, 4). Interestingly, expression levels of the *CD161* gene were significantly lower in progressive MS compared to the healthy population (Figure 1A). Considering that this marker may be expressed by distinct immune cell subsets, including T lymphocytes and natural killer (NK) cells, we checked CD161 protein levels in PBMC from a new cohort of sex- and age-matched healthy and primary progressive MS subjects (Supplementary Table 1) by multiparametric flow cytometry. As shown in Figure 1B, the overall frequency of CD3 positive T lymphocytes, CD19 positive B lymphocytes and CD56 positive NK cells did not differ between healthy and diseased individuals. Similarly, the overall frequencies of CD161 expressing T cells, B lymphocytes and NK cells were comparable in the two groups of subjects (Figure 1B). When analyzing separately CD4 and CD8 T lymphocytes and stratifying them in naïve (CD45RO negative), central memory (CM, CD45RO and CCR7 positive) and effector memory (EM, CD45RO positive and CCR7 negative) T lymphocytes, additional differences appeared in the percentage of distinct CD161-expressing T cells in the two study groups. In fact, in primary progressive MS we detected a trend to higher frequency of CD161+ CD4 T cells (Mean ± SEM Ctrl vs. PP-MS 19.86 ± 1.29 vs. 25.44 ± 2.35, *p*-value 0.05) that was reproduced in the naïve CD4 T cell population (Mean ± SEM Ctrl vs. PP-MS 3.37 ± 0.49 vs. 9.26 ± 1.97, *p*-value 0.0041) and an overall lower percentage of CD161 expressing CD8 T cells (Mean ± SEM Ctrl vs. PP-MS 19.85 ± 1.57 vs. PP-MS: 14.82 ± 1.62, *p*-value 0.046) which was paralleled by 32.5% reduction in the CD161 expressing effector memory CD8 T cell population (Mean ± SEM Ctrl vs. PP-MS 39.92 ± 4.87 vs. 27.08 ± 3.60, *p*-value 0.043, Figure 1C).

**Figure 2:**
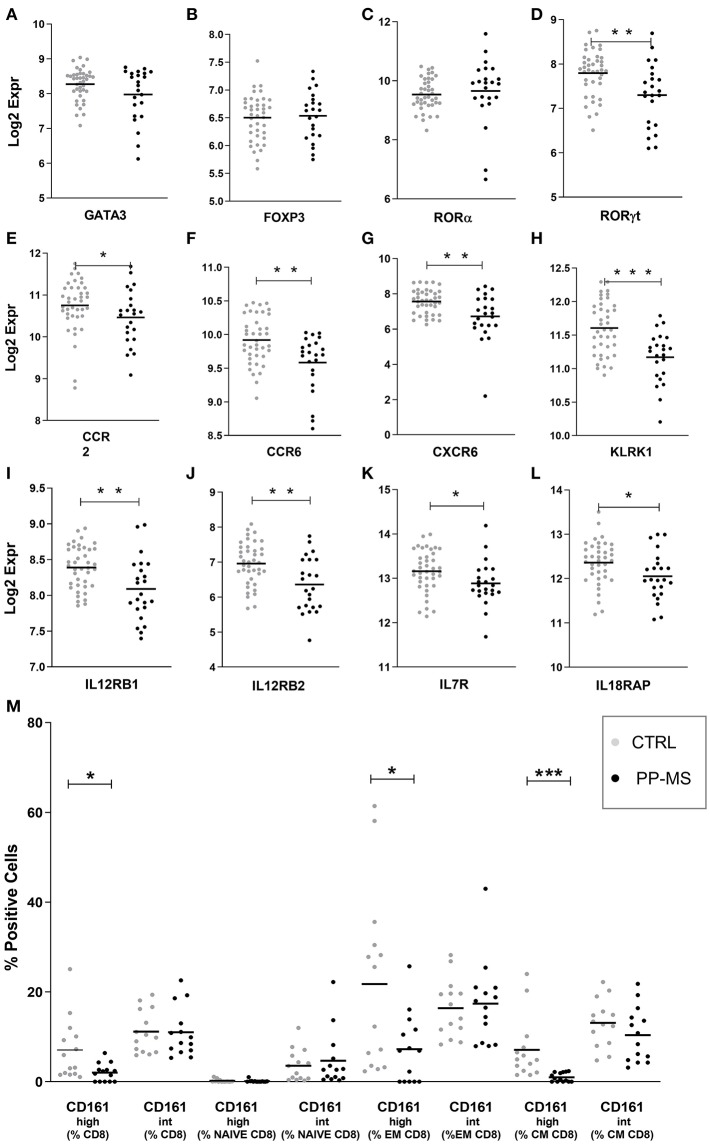
**(A–L)** Transcript levels of T cell-related transcription factors **(A–D)** and MAIT cell markers **(E–L)** in PBMC of the first cohort of healthy and PP-MS subjects. **(M)** Frequency of circulating CD161^high^ and CD161^int^ cells among naïve, EM and CM CD8 T lymphocytes in the second case-control cohort. ^*^p < 0.05, ^**^p < 0.01, ^***^p < 0.001.

We then queried our transcriptomics dataset for the expression of transcription factors regulating T cell differentiation, activation and maintenance. While the probe for Tbet/TBX21 [implied in Th1 differentiation and CD8 T cell effector function (12)] did not display detectable signals to be analyzed, those for GATA-3 [implied in Th2 differentiation and CD8 T cell survival, (12, 13)], Foxp3 [expressed by CD4 and CD8 regulatory T cells (14, 15) and RORA [a master gene for IL17 production (16, 17)], displayed equal expression in PBMC of healthy and PP-MS subjects (Figures 2A–C). Differently, the transcript for RORγt, another transcription factor characterizing IL17-producing T cells (12, 18), was significantly lower in PBMC from PP-MS compared to those from healthy donors (Figure 2D). CD8 T lymphocytes capable of secreting IL17 display high levels of CD161 and RORγt, and include the mucosal-associated invariant T (MAIT) cell subset (19, 20). We then verified mRNA levels of other MAIT cell markers, such as CCR2, CCR6, CXCR6, IL12 receptor, IL7 receptor and KLRK1/NKG2D (21), and found reduced transcripts for all of these genes in mononuclear cells of PP-MS compared to those of control subjects (Figures 2E–L). These transcriptional observations suggest the specific reduction of the CD8+ CD161^high^ T cell population in progressive multiple sclerosis. To verify this hypothesis we stratified the multiparametric flow cytometry data according to the intensity of CD161 protein on cell membrane of CD8 T cells and compared the percentage of CD161^high^ or intermediate (^int^) CD8 T cell subsets in progressive MS and control subjects. As shown in Figure 2M, while the frequency of CD8+ CD161^int^ T cell subsets did not differ in the two study groups, that of CD161^high^ T cells among all CD8 T cells was strongly reduced or completely lost in progressive MS (Mean ± SEM Ctrl vs. PP-MS 7.07 ± 1.83 vs. 2.03 ± 0.53, *p*-value 0.02) and this evidence was reproduced in the effector and central memory CD8 T cell compartment (Mean ± SEM Ctrl vs. PP-MS, EM 21.78 ± 5.34 vs. 7.24 ± 2.07, *p*-value 0.02; CM 7.10 ± 1.83 vs. 0.99 ± 0.26, *p*-value 0.0001). Overall, these data demonstrate the selective loss of circulating CD8+ CD161^high^ T cells in primary progressive multiple sclerosis.

## Discussion

Here we report alterations in the frequency of circulating CD161-expressing T cells in primary progressive multiple sclerosis.

The first observation is the higher frequency of CD161+ naïve CD4 T cells in PP-MS compared to the heathy subjects. Considering that all IL17-producing T lymphocytes originate from CD161+ naïve CD4 T cells (22), this finding suggests larger potential of generation of proinflammatory Th17 T cells under disease.

The second observation regards the lower frequency of CD161+ memory CD8 T lymphocytes which is selectively limited to those cells expressing CD161 protein at high levels. A few studies investigated the frequency of circulating CD8+ CD161^high^ T cells in MS subjects, with contradictory results in relapsing-remitting MS or pooled cohorts of MS patients with different disease courses (9, 23–25). In this respect, our study represents the first description of the selective loss of circulating CD8+ CD161^high^ T cells in primary progressive MS. Most of the CD8+ CD161^high^ T cells are MAIT cells, an IL17-producing T cell population with an invariant T cell receptor alpha chain (Vα7.2) recognizing microbial products in the context of the non-polymorphic MHC-related protein 1 (21). Interestingly, MAIT cells may downregulate CD161, as shown after HIV infection (26), thus the loss in CD8+ CD161^high^ T cells may not necessarily reflect a lower frequency of MAIT cells. On the other hand, the concomitant transcriptional downregulation of several MAIT markers as detected in PBMC from PP-MS supports the hypothesis of the overall loss of circulating MAIT cells, an issue which remains open for further investigation. In subjects with chronic infections, inflammatory disorders or autoimmune diseases the frequency of CD8+ CD161+ T lymphocytes is reduced in blood (21, 27–29), while enriched in infected or inflamed tissue (21), suggesting relocation of these cells from blood to inflamed site. Notably, the observations that CD161-expressing CD8+ or Vα 7.2+ T cells infiltrate MS lesions and bear an inflammatory phenotype (9, 24, 25), and that autologous hematopoietic stem cell transplantation in subjects with aggressive, highly inflammatory MS depletes circulating MAIT cells for several years (30), support the hypothesis of a pathogenic role for these cells in MS. On the contrary, evidences in the animal model of MS suggest a protective action of MAIT cells during neuroinflammation (31). Differently from relapsing-remitting MS, primary progressive MS is not characterized by frequent inflammatory waves toward the CNS parenchyma, so whether and where the CD8+ CD161^high^ T cells or specifically MAIT cells relocate in PP-MS is unknown. Interestingly, ectopic lymphoid follicles may form in the meninges of MS patients, including progressive cases (32), and CD161+ CD8+ T cells have been found in meningeal B-cell follicles (9). However, whether these cells are indeed MAIT cells remains to be established.

## Ethics Statement

Investigations were conducted according to the principles expressed in the Declaration of Helsinki and after approval of the study by the Ethics Committee of Ospedale San Raffaele. Peripheral blood was drawn after signing of the informed consent.

## Author Contributions

MA performed transcriptomics and statistical analyses and wrote the paper. CB and NS performed and analyzed flow cytometry experiments. GD, MR, FS, BC, LM, and VM enrolled the patients for the study and provided clinical information. GC critically discussed the project and the results. CF conceived and designed the experiments, coordinated the study, discussed the results and wrote the paper.

### Conflict of Interest Statement

MR received honoraria from Genzyme and Merck-Serono. FS received speaker honoraria an/or travel grants from Biogen, Merck, Novartis, Sanofi-Genzyme and Teva, outside the submitted work. LM received honoraria for consultancy, participation to advisory boards and scientific meetings from Biogen, Genzyme, Merck, Novartis, Teva, Roche. VM received honoraria for consultancy and travel grants for congress participation from Biogen-Idec, Merck, Novartis, Genzyme, Almirall and Teva. GC received honoraria for consulting services and/or speaking activities from Novartis, Teva, Sanofi Genzyme, Merck, Biogen, Roche, Almirall, Celgene, Forward Pharma, Medday and Excemed. CF received research support from Merck-Serono, Teva, Novartis, Italian Ministry of Health and Fondazione Italiana Sclerosi Multipla. The remaining authors declare that the research was conducted in the absence of any commercial or financial relationships that could be construed as a potential conflict of interest.

## Supplementary Material

Supplementary Figure 1 Gating strategy and representative results. Thresholds were set on FMO and isotype controls.

Supplementary Table 1 Clinical and demographic features of the validation cohort.
