# Supplementary figures and images for: Loss of Circulating CD8+ CD161high T Cells in Primary Progressive Multiple Sclerosis
Source: Front Immunol. 2019 Aug 14;10:1922. doi: 10.3389/fimmu.2019.01922 (PMC6702304; doi:10.3389/fimmu.2019.01922)

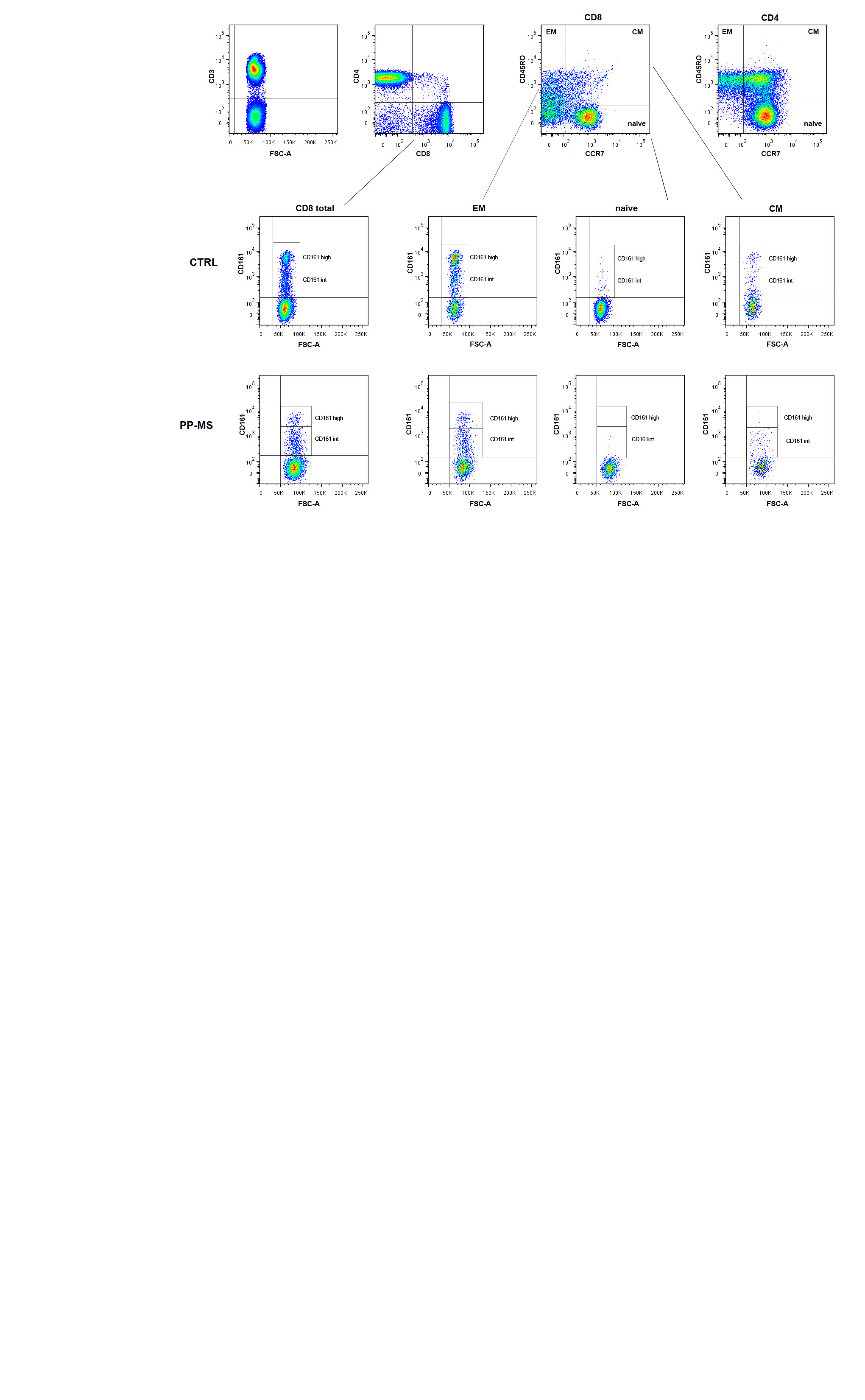

Supplement: Supplementary Figure 1 — Gating strategy and representative results. Thresholds were set on FMO and isotype controls. [file Image_1.JPEG]
