# Supplementary material for: Loss of Circulating CD8+ CD161high T Cells in Primary Progressive Multiple Sclerosis
Source: Front Immunol. 2019 Aug 14;10:1922. doi: 10.3389/fimmu.2019.01922 (PMC6702304; doi:10.3389/fimmu.2019.01922)
Supplement: Supplementary Table 1 — Clinical and demographic features of the validation cohort. [file Table_1.pdf]

Supplementary Table 1. Clinical and demographic features of the validation cohort

	HC	PP-MS
**# (female, male)**	14 (12, 2)	14 (12, 2)
**Age, y**	46,1 ± 9,7	49,1 ± 10,8
**Disease duration, y**	n.a.	5,2 ± 4,6
**EDSS score**	n.a.	4,9 ± 1,1

HC, healthy controls; PP-MS, primary progressive MS; y, years; n.a., not applicable
